# Study of the antimicrobial activity of cationic carbosilane dendrimers against clinical strains of multidrug-resistant bacteria and their biofilms

**DOI:** 10.3389/fcimb.2023.1203991

**Published:** 2023-10-10

**Authors:** Marcos Hernando-Gozalo, John Jairo Aguilera-Correa, Carlos Rescalvo-Casas, Laura Seijas-Pereda, Carlos García-Bertolín, Francisco Javier de la Mata, Javier Sánchez-Nieves, Juan Cuadros, Ramón Pérez-Tanoira

**Affiliations:** ^1^ University of Alcalá, Department of Organic and Inorganic Chemistry, Research Institute in Chemistry “Andrés M. del Río” (IQAR), Madrid, Spain; ^2^ Clinical Microbiology Department, Instituto de Investigación Sanitaria (IIS)-Fundacion Jimenez Diaz-Universidad Autónoma de Madrid (UAM), Madrid, Spain; ^3^ Centro de Investigación Biomédica en Red de Enfermedades Infecciosas (CIBERINFEC), Instituto de Salud Carlos III, Madrid, Spain; ^4^ University of Alcalá, Department of Biomedicine and Biotechnology, Faculty of Medicine, Madrid, Spain; ^5^ Clinical Microbiology Department, Hospital Universitario Príncipe de Asturias, Alcalá de Henares, Spain; ^6^ Ramón y Cajal Institute for Health Research, Ramón y Cajal Health Research Institute (IRYCIS), Madrid, Spain; ^7^ Networking Research Center on Bioengineering, Biomaterials and Nanomedicine (CIBER-BBN), Madrid, Spain

**Keywords:** multiresistant bacteria, biofilms, antibacterial, carbosilane dendrimers and dendrons, quaternary ammonium salts

## Abstract

**Introduction:**

Antimicrobial Resistance is a serious public health problem, which is aggravated by the ability of the microorganisms to form biofilms. Therefore, new therapeutic strategies need to be found, one of them being the use of cationic dendritic systems (dendrimers and dendrons).

**Methods:**

The aim of this study is to analyze the *in vitro* antimicrobial efficacy of six cationic carbosilane (CBS) dendrimers and one dendron with peripheral ammonium groups against multidrug-resistant bacteria, some of them isolated hospital strains, and their biofilms. For this purpose, minimum inhibitory concentration (MIC), minimum bactericidal concentration (MBC), minimum biofilm inhibitory concentration (MBIC) and minimum eradication biofilm concentration (MBEC) studies were carried out. In addition, the cytotoxicity on Hela cells of those compounds that proved to be the most effective was analyzed.

**Results:**

All the tested compounds showed *in vitro* activity against the planktonic forms of methicillin-resistant *Staphylococcus aureus* and only the dendrimers BDSQ017, BDAC-001 and BDLS-001 and the dendron BDEF-130 against their biofilms. On the other hand, only the dendrimers BDAC 001, BDLS-001 and BDJS-049 and the dendron BDEF-130 were antibacterial in vitro against the planktonic forms of multidrug-resistant *Pseudomonas aeruginosa*, but they lacked activity against their preformed biofilms. In addition, the dendrimers BDAC-001, BDLS-001 and BDSQ-017 and the dendron BDEF-130 exhibited a good profile of cytotoxicity *in vitro*.

**Discussion:**

Our study demonstrates the possibility of using the four compounds mentioned above as possible topical antimicrobials against the clinical and reference strains of multidrug-resistant bacteria.

## 1 Introduction

The Antimicrobial Resistance (AMR) is a serious public health problem, recognized by the World Health Organization (WHO), associated with relevant socioeconomic consequences (Gil-Gil et al., 2019). Currently, AMR causes about 700,000 deaths worldwide annually, but this is expected to increase to 10 million by 2050 (Dadgostar, 2019). Consequently, it is possible that an AMR pandemic may occur around that year, if effective and robust measures are not taken against it.

The microorganisms can exist in two forms: in planktonic or free-living form or in biofilms (Verderosa et al., 2019). These are conglomerates of microbial cells of at least one species that are irreversibly associated or not to a surface or an interface, and embedded in a self-produced matrix of polymeric extracellular substances, where sociomicrobiological interactions prevail (Costerton et al., 1999; Parsek and Greenberg, 2005). Currently, it is estimated that 80% of human infections are due to biofilms such as skin and soft tissue infections (vaginitis, conjunctivitis, otitis or chronic wounds) and those associated with implanted medical devices (Verderosa et al., 2019). In addition, the biofilms offer protection to their constituent cells from the immune system and they hinder the penetration of antibiotics, making microorganisms above a thousand times more tolerant to them (Verderosa et al., 2019). Therefore, finding new strategies against multidrug-resistant microorganisms and their biofilms is urgent.

One of the promising strategies is the use of quaternary ammonium salts, which show antibacterial activity. Their mechanism of action is due to the displacement of divalent cations from the bacterial membrane, inducing their destabilization and subsequent lysis (Fuentes-Paniagua et al., 2016). Therefore, their incorporation into various polymers originates polycationic systems with higher charged surface areas and antimicrobial activities than the monofunctional systems. Moreover, they show advantages such as hydrosolubility, non-volatility, chemical stability, long-term antimicrobial activity and the possibility of introducing different modifications to improve their behaviour (Fuentes-Paniagua et al., 2016).

Cationic dendrimers are a particular system with quaternary ammonium salts integrated into their structures. These are complex synthetic macromolecules with hyperbranched structures, nanometric size and a highly controlled three-dimensional shape. They are usually spherical, with a central core, some repetitive branching and a surface with multiple functional groups (Ortega et al., 2020). Related systems are dendrons, which are conical molecules with an extra active residue, the focal point, which can be used to attach a second functional group (Fuentes-Paniagua et al., 2016).

There are multiple cationic dendrimers with antibacterial properties *per se* against Gram-positive and Gram-negative bacteria, most notably poly(amidoamine) (PAMAM) (Gholami et al., 2017), poly(propylenimine) (PPI) (Chen et al., 2000), phosphor-viologens, poly(propyleneoxide) amines and carbosilane (CBS) (de la Mata et al., 2022). In particular, the CBS dendritic macromolecules present a common structure based on carbon-carbon and carbon-silicon bonds. In turn, the tetravalent silicon atom allows different degrees of branching of the dendritic system and plays a relevant role in the solubility and the polarity of these compounds. Therefore, the CBS dendrimers are very stable, flexible, inert and hydrophobic macromolecules, being very attractive for biomedical applications (de la Mata et al., 2022).

Numerous studies have demonstrated *in vitro* antibacterial efficacy of cationic CBS dendrimers and dendrons with peripheral ammonium groups against Gram-positive and Gram-negative bacteria (Fuentes-Paniagua et al., 2016; Heredero-Bermejo et al., 2018; Quintana-Sanchez et al., 2022), and some of them against their biofilms (Barrios-Gumiel et al., 2019). However, these studies have been performed mainly against laboratory strains, which show lower virulence than clinical strains. In fact, nosocomial infections have become also a major concern inside health-care facilities, so it would be interesting to test these compounds against the clinical strains and their biofilms.

Thus, the aim of this study is to demonstrate *in vitro* antimicrobial efficacy of six cationic CBS dendrimers and one dendron with peripheral ammonium groups against clinical strains of multidrug-resistant bacteria and their biofilms, analyzing the influence of the type of bacteria, the dendrimer core and the cationic group (ammonium, guanidinium and trimethylammonium) on such efficacy.

## 2 Materials and methods

### 2.1 Antimicrobial compouds

**Figure 1:**
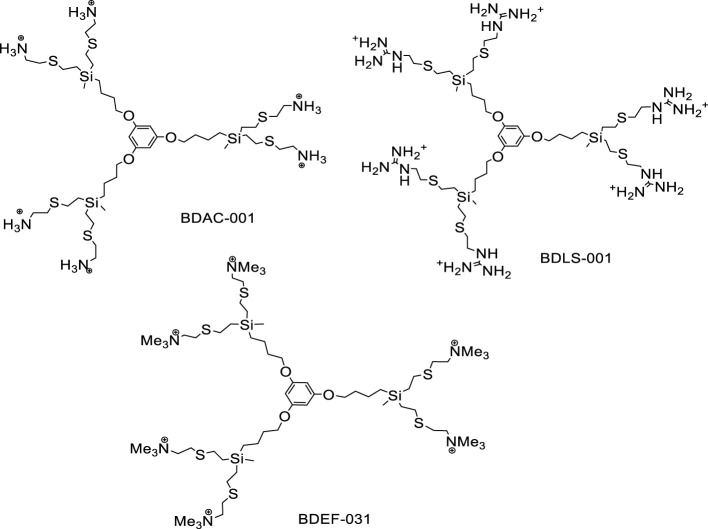
Structure of CBS cationic dendrimers with phloroglucinol core BDAC-001 (G_1_O_3_(S-NH_3_^+^)_6_), BDLS-001 (G_1_O_3_(S-GU^+^)_6_) and BDEF-031 (G_1_O_3_(S-NMe_3_^+^)_6_).

**Figure 2:**
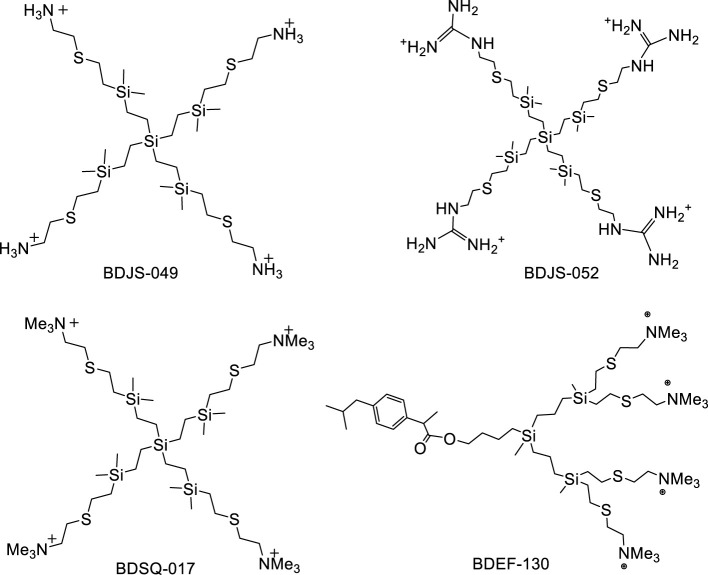
Structure of CBS cationic dendrimers with a silicon atom core BDJS-049 (G_0_Si(SiMe_2_-NH_3_^+^)_4_), BDJS-052 (G_0_Si(SiMe_2_-GU^+^)_4_) and BDSQ-017 (G_0_Si(SiMe_2_-NMe_3_^+^)_4_), and cationic dendron BDEF-130 (IbuG_2_(S-NMe_3_^+^)_4_).

The nomenclature in this article for the phloroglucinol-core dendrimers (1,3,5-trihydroxybenzene) is G_n_O_3_(S-Y) _m_ type, for the silicon-core dendrimers is G_n_Si(SiMe_2_-Y)_m_ type and for the dendron is XG_n_(S-Y)_m_ (**Figures 1**, **2**). These describes their structure as follows: G_n_ corresponds to the carbosilane framework and to the generation (n); O_3_ and Si describe the core, phloroglucinol (1,3,5-C_6_H_6_O_3_) or silicon atom, respectively; X means the focal point of the dendron, and (S-Y)_m_ and (SiMe_2_-Y)_m_ indicate the type of outlying groups (Y), their number (m) and the presences of a sulfur atom near the surface in the dendrimers with phloroglucinol core and in the dendron, and of a SiMe_2_ group in those with a silicon atom core, according to the synthetic methodology described previously (Fuentes-Paniagua et al., 2014; Heredero-Bermejo et al., 2016; Perisé-Barrios et al., 2016; Quintana-Sanchez et al., 2022).

We chose to analyze the antimicrobial properties of the dendrimers BDAC-001, BDLS-001, BDEF-031 and BDSQ-017 because they had demonstrated *in vitro* good antibacterial efficacy against laboratory strains in previous studies by our group (Fuentes-Paniagua et al., 2014; Fuentes-Paniagua et al., 2016; Heredero-Bermejo et al., 2018; Quintana-Sanchez et al., 2022) and we wanted to know whether they also maintained such efficacy against multidrug-resistant clinical strains. In addition, we included the recently synthesized dendrimers BDJS-049 and BDJS-052 to describe their microbicidal properties. Consequently, by having two groups of dendrimers with different cores (phloroglucinol ring or silicon atom) and peripheral groups (ammonium, guanidinium and trimethylammonium), a global view about the influence of the core and the peripheral groups on the antibacterial efficacy could be obtained.

On the other hand, dendrons have a focal point to which several ligands, such as metal atoms or small molecules, can be attached. This generates systems that can exhibit different properties both at the peripheral groups and at the focal point. In particular, the dendron BDEF-130 combines the antibacterial properties of the trimethylammonium peripheral groups with the anti-inflammatory properties of the ibuprofen group at the focal point (Fuentes-Paniagua et al., 2016; Perisé-Barrios et al., 2016). This combination could be very interesting for the development of possible topical antimicrobials to promote healing of chronic infected wounds. For this reason we also chose this compound, which had already demonstrated *in vitro* anti-inflammatory efficacy (Perisé-Barrios et al., 2016).

Since BDJS-049 and BDJS-052 are newly synthesized compounds, their chemical synthesis is explained next. All reactions were carried out under inert atmosphere and solvents were purified from appropriate drying agents when necessary. Thiol-ene reactions were carried out employing a novaLIGHT TQ150-Z0 Lamp from UV-Consulting Peschl with maximum energy at 365 nm, in normal glassware under inert atmosphere. Reagents, unless otherwise stated, were obtained from commercial sources and used as received. The NMR spectra (Nuclear Magnetic Resonances) were recorded on Bruker Advance Neo 400 at ambient temperature (400.13 MHz (^1^H), 100.60 MHz (^13^C)). The elemental analyses were carried out on a PerkinElmer 240C instrument.

#### Compound BDJS-049

Compound Si(Si-V)_4_ [13] (0.750 g, 1.56 mmol) was dissolved in THF/MeOH (1:2) and 2-aminoethanethiol hydrochloride (0.70 g, 6.2 mmol) and 2,2-dimethoxy-2-phenylacetophenone (DMPA) (4% mol, 0.064 g) were added in two portions, one half at the beginning of the reaction and the second half after 2 h. of reaction. The reaction mixture was deoxygenated and stirred under ultraviolet irradiation for a total time of 4 h. Then, reaction was checked by ^1^H-NMR to confirm its finalization. Afterwards, volatiles were removed by rotatory evaporation and the remaining solid was washed with MeOH/Et_2_O. Compound **BDJS-049** was obtained as a pale yellow solid (1.21 g, 83%). Data for **BDJS-049**: ^1^H-NMR (MeOH-d4): δ = 0.07 (s, 24 H, SiMe_2_), 0.50 (m, 16 H, SiCH_2_CH_2_Si, SiCH_2_CH_2_Si), 0.95 (m, 8 H, SiCH_2_CH_2_S), 2.67 (m, 8 H, SiCH_2_CH_2_S), 2.87 (s, 8 H, SCH_2_CH_2_N), 3.16 (m, 8 H, SCH_2_CH_2_N). ^13^C{H} NMR: δ = −5.2 (SiMe_2_), 2.2 (SiCH_2_CH_2_Si), 7.0 (SiCH_2_CH_2_Si), 14.9 (SiCH_2_CH_2_S), 26.8 (SiCH_2_CH_2_S), 28.2 (SCH_2_CH_2_N), 38.5 (SCH_2_CH_2_N). Elemental analysis for C_32_H_34_Cl_4_N_4_S_4_Si_5_ (935.52 g/mol): Calc. C, 41.08; H, 9.05; N, 5.99; S, 13.71; Obt. C, 41.73; H, 9.73; N, 5.81; S, 13.48.

#### Compound BDJS-052

Compound **BDJS-049** (0.500 g, 0.534 mmol) and 1H-pyrazole-1-carboxamidine hydrochloride (0.470 g, 3.20 mmol) in the presence of diisopropylethylamine (DIPEA) (6.40 mmol) were heated in EtOH (25 mL) at 55°C during 16 h under inert atmosphere. Afterwards, volatiles were removed under vacuum and the residue was solved in MeOH and precipitated with acetone, yielding **BDJS-052** as a yellow solid (0.466 g, 79%). Data for **BDJS-052**: ^1^H-NMR (MeOH-d4): δ = 0.07 (s, 24 H, SiMe_2_), 0.48 (m, 16 H, SiCH_2_CH_2_Si, SiCH_2_CH_2_Si), 0.93 (m, 8 H, SiCH_2_CH_2_S), 2.67 (m, 8 H, SiCH_2_CH_2_S), 2.78 (s, 8 H, SCH_2_CH_2_N), 3.43 (m, 8 H, SCH_2_CH_2_N). ^13^C{H} NMR: δ = −3.9 (SiMe_2_), 3.1 (SiCH_2_CH_2_Si), 8.2 (SiCH_2_CH_2_Si), 16.2 (SiCH_2_CH_2_S), 28.1 (SiCH_2_CH_2_S), 31.3 (SCH_2_CH_2_N), 41.73 (SCH_2_CH_2_N), 159.0 (NHC(NH_2_). Elemental analysis for C_36_H_92_Cl_4_N_12_S_4_Si_5_ 1103.68 g/mol): Calc. C, 39.18; H, 8.40; N, 15.23; S, 11.62; Obt. C, 38.29; H, 8.02; N, 15.01; S, 12.40.

### 2.2 Microbiological studies

The antimicrobial capabilities of these compounds were first tested against the biofilm-formation collection strains of *Pseudomonas aeruginosa* ATCC 27853 and *Staphylococcus aureus* ATCC 29213. Then, they were evaluated against clinical strains of methicillin-resistant *Staphylococcus aureus* (MRSA 1 and MRSA 2) and multidrug-resistant *Pseudomonas aeruginosa* (PA 24 and PA 35) isolated from patients diagnosed by the Clinical Microbiology Department of the Jiménez Díaz Foundation University Hospital (Madrid, Spain) (Aguilera-Correa et al., 2021). Specifically, MRSA 1 was isolated from the infected wound of a 73-year-old man and MRSA 2 from the paronychia of a 92-year-old male, while PA 24 and PA 35 were isolated from the sputum of a 67-year-old man and a 57-year-old woman, respectively. All strains were kept frozen at -80°C until studies were performed. The clinical strains are positive for biofilm formation. The strains were defrosted, seeded on colistin-nalidixic acid agar (CNA) for *S. aureus* and on McConkey agar for *P. aeruginosa* and they were incubated at 37°C for 24h.

#### 2.2.1 Antibiotic susceptibility studies

The antibiotic susceptibility of the above six strains was determined using VITEK 2 Systems (Biomérieux, Îlle de France, France).

#### 2.2.2 Minimum inhibitory concentration and minimum bactericidal concentration

The minimum inhibitory concentration (MIC) was determined according to the standardized broth microdilution method of the Clinical and Laboratory Standards Institute (CLSI, 2018). A bacterial inoculum was prepared with a bacterial concentration of 1x10^8^ colony-forming units per milliliter (CFU/mL), corresponding to 0.50 ± 0.02 of the McFarland turbidity standard, and diluted 1:100 in Müller-Hinton broth (MHB). In a rounded-bottom 96-well plate (Thermo Fisher Scientific, Roskilde, Denmark), a series of double microdilutions of dendrimers and dendrons, in the concentration range of 1024-1 mg/L, and a bacterial inoculum at the concentration of 1x10^6^ CFU/mL were added. It was statically incubated for 24 h at 37°C and 5% CO_2_ and the MIC was determined visually as the lowest concentration of antimicrobial at which no bacterial growth was observed.

The minimum bactericidal concentration (MBC) was determined by the flash microbicidal method (Hernandes et al., 2013). For this, 180 μL of tryptose soy broth (TSB) and 20 μL of the previous MIC plate were dispensed to each well of a flat-bottomed 96-well plate. The plate was statically incubated at 37°C and 5% CO_2_ for 24 h and the MBC was visually determined as the lowest concentration of antimicrobial at which no bacterial growth was observed. The experiments were performed in quadruplicate (n=4).

#### 2.2.3 Minimum biofilm inhibitory concentration and minimum biofilm eradication concentration

The minimum biofilm inhibitory concentration is the minimum concentration of an antimicrobial compound needed to inhibit the visible growth of a bacterial biofilm, whereas the minimum biofilm eradication concentration (MBEC) is the minimum concentration of an antimicrobial compound required to kill a bacterial biofilm (Nam-Cha et al., 2023). As *P. aeruginosa* is mobile and *S. aureus* is immobile, two different protocols are used: (Ceri et al., 1999) for the former and (Pettit et al., 2005) for the latter, to determine the MBIC and MBEC.

Regarding the MBIC of the compounds against *P. aeruginosa*, the biofilm formation was induced on the pegs of the MBEC™ Biofilm Inoculator device (Innovotech Inc, Edmonton, AB, Canada) by inoculating 200 μL of TSB, with a bacterial concentration of 1x10^6^ CFU/mL per well in a 96-well flat-bottom plate. This was statically incubated for 24 h at 37°C and 5% CO_2_. The next day, the device pegs were washed twice with 200 μL/well of 0.9% saline to remove planktonic bacteria. They were then brought to a flat-bottom 96-well plate with a series of double microdilutions of dendrimers and dendrons, in a concentration range of 1024-1 mg/L, in MHB to a final volume of 200 μL/well. This plate was statically incubated for 24 h at 37°C and 5% CO_2_ and the next day, the MBIC of the compounds was determined by measuring the absorbance at 600 nm in a microtiter plate reader (Epoch™, BioTek Instruments, Winooski, VT, USA). This was accompanied by a series of double microdilutions of the dendrimers and dendrons without bacteria as a negative absorbance control.

For the measurement of MBEC, the pegs of the MBIC plate were washed twice with 200 μL/well of 0.9% saline and placed on another flat-bottom plate with 200 μL/well of TSB. Such plate was statically incubated for 24 h at 37°C and 5% CO_2_ and finally, the MBEC of the compounds was established by measuring the absorbance at 600 nm. These experiments were carried out in quadruplicate (n=4).

For MBIC of the compounds against *S. aureus*, 100 μL/well of MHB, at a bacterial concentration of 1x10^6^ CFU/mL, was dispensed into a 96-well flat-bottom plate to promote biofilm formation. The plate was incubated statically for 24 h at 37°C and 5% CO_2_. Then, the supernatant was aspirated from each well and 200 μL/well of a series of double microdilutions of dendrimers and dendrons in MHB were added at a concentration range of 1024-1 mg/L. This plate was incubated for 24 h at 37°C and 5% CO_2_ and after that, the MBIC of the compounds were obtained by measuring the absorbance at 600 nm in a microtiter plate reader. The latter was accompanied by a series of double microdilutions of the dendrimers and dendrons without bacteria as a negative absorbance control.

For MBEC, the bottom of the wells of the MBIC plate was scraped with the aid of a 100 μL pipette tip and 20 μL was taken and carried to a new plate to which 180 μL/well of TSB had been added. The plate was incubated statically for 24 h at 37°C and 5% CO_2_ and the MBEC of these compounds were calculated by measuring the absorbance at 600 nm. These experiments were performed in quadruplicate (n=4).

### 2.3 Citotoxicity assays on HeLa cells

The cytotoxicity assays were performed on HeLa cells (cervical cancer, ATCC^®^ CCL-2™, Manassas, Virginia, USA). For this purpose, the cells were cultured in Dulbecco’s Modified Eagle Medium (DMEM) (Gibco, Thermo-Fisher Scientific, Waltham, MA, USA) supplemented with 10% fetal bovine serum (Sigma-Aldrich Ltd.) and 1% antibiotic mixture. The cells were then seeded in 24-well plates (Greiner Bio-One) at a density of 8000 cells per well and incubated for 72 h in an atmosphere of 37°C, 5% CO_2_ and 95% humidity until a confluent monolayer was achieved.

After incubation, the culture medium was discarded and 450 µL of fresh medium and 50 µL of the antimicrobial compounds prepared in phosphate-buffered saline (PBS) (Sigma Aldrich, St. Louis, MO, USA), in the concentration range of 200-0.1 µM, were added to each well. The control wells received fresh medium instead of dendrimers and dendrons. These plates were incubated for 24 h under the above conditions.

To assess the cytotoxicity of these compounds, MTT (3-(4,5-dimethylthiazol-2-yl)-2,5-diphenyltetrazolium bromide) colorimetric viability assay was performed, based on the reduction of the MTT molecule to formazan when exposed to viable cells, according to the protocol of (Kumar et al., 2018). For this, the culture medium was discarded again and 500 µL of fresh medium and 50 µL of MTT (Sigma-Aldrich Ltd.) at a concentration of 12 mM were added to each well. After incubation for 4h at 37°C, the MTT was removed, 500 µL of dimethyl sulfoxide (DMSO) were added to dissolve the formazan crystals and the absorbance of the plates was measured in a microplate absorbance reader at 570-630 nm (BioTek Instruments Inc. Model: ELX 800). The assays were performed in triplicate (n=3).

The cell viability was calculated according to this formula: %Cell viability = (A_S_/A_C_) x 100, where A_S_ is the absorbance of the sample and A_C_ is the absorbance of the control. In addition, the half-maximal inhibitory concentration 50 (IC_50_), which is the concentration of a compound necessary to reduce the growth of a cell population *in vitro* by 50%, was calculated with AAT Bioquest, Inc. (Quest Graph ™ IC50 Calculator). AAT Bioquest. https://www.aatbio.com/tools/ic50-calculator (accesed on 5^th^ July 2023).

### 2.4 Statistical analysis

All the exposed data of concentrations are expressed as medians and interquartile ranges (IQR). Cytotoxicity values of the compounds are represented as means and standard deviation (SD). Calculations were made using Microsoft Excel 2016. Statistical tests were performed using SPSS v20.0 (IBM Corp., Armonk, NY, USA.). Data were evaluated using a Student’s t-test and statistical significance was set at p-values ≤0.05.

## 3 Results

### 3.1 Susceptibility studies

The antibiograms of the *S. aureus* and *P. aeruginosa* strains used in this study were showed in **Tables 1**, **2**. With respect to *S. aureus* (**Table 1**), both clinical strains were resistant to the beta-lactam antibiotics. On the other hand (**Table 2**), PA 24 presented high level cephalosporinases and was resistant to the carbapenem antibiotics, while PA 35 strain also showed extended-spectrum beta-lactamases.

**Table 1:** Antibiogram of *Staphylococcus aureus* strains used in this study.

Antibiotic	ATCC 29213 (MIC)	MRSA 1 (MIC)	MRSA 2 (MIC)
Cefoxitin detection	NEG	POS	POS
Benzylpenicillin	R (≥ 0.5)	R (≥ 0.5)	R (≥ 0.5)
Oxacillin	S (≤ 0.25)	R (≥ 4)	R (≥ 4)
Ceftaroline	S (0.25)	R (0.5)	S (0.25)
Gentamycin	S (≤ 0.5)	S (≤ 0.5)	S (≤ 0.5)
Tobramycin	S (≤ 1)	R (≥ 16)	S (≤ 1)
Levofloxacin	I (0.25)	R (≥ 8)	R (≥ 8)
Inducible resistance to clindamycin	NEG	NEG	NEG
Erythromycin	S (1)	R (≥ 8)	R (≥ 8)
Clindamycin	S (0.25)	R (≥ 8)	S (0.25)
Linezolid	S (2)	S (1)	S (2)
Daptomycin	S (0.25)	S (0.25)	S (0.25)
Teicoplanin	S (≤ 0.5)	S (≤ 0.5)	S (≤ 0.5)
Vancomycin	S (1)	S (1)	S (≤ 0.5)
Tigecycline	S (≤ 0.12)	S (≤ 0.12)	S (≤ 0.12)
Fosfomycin	S (≤ 8)	S (≤ 8)	S (≤ 8)
Mupirocin	S (≤ 1)	S (≤ 1)	R (≥ 512)
Rifampicin	S (≤ 0.03)	S (≤ 0.03)	S (≤ 0.03)
Trimethoprim/Sulfamethoxazole	S (≤ 10)	S (≤ 10)	S (≤ 10)

MIC, Minimum Inhibitory Concentration (mg/L); S, susceptible; I, intermediate; R, resistant.

**Table 2:** Antibiogram of *Pseudomonas aeruginosa* strains used in this study.

Antibiotic	ATCC 27853 (MIC)	PA 24 (MIC)	PA 35 (MIC)
Piperacillin/Tazobactam	I (≤ 4)	R (≥ 128)	R (≥ 128)
Ceftazidime	I (2)	R (16)	R (≥ 64)
Ceftolozane/Tazobactam	S (0.5)	S (1)	S (2)
Cefepime	I (2)	R (16)	R (16)
Aztreonam	I (2)	*R (16)	R (32)
Imipenem	I (2)	R (≥ 16)	R (≥ 16)
Meropenem	S (≤ 0.25)	I (8)	R (≥ 16)
Amikacin	S (2)	S (4)	S (4)
Gentamycin	S (≤ 1)	R (≥ 16)	R (≥ 16)
Tobramycin	S (≤ 1)	R (≥ 16)	R (≥ 16)
Ciprofloxacin	I (≤ 0.25)	R (≥ 4)	R (≥ 4)
Fosfomycin	S (≤ 16)	(≥ 256)	(≥ 256)
Colistin	S (2)	S (≤ 0.5)	S (≤ 0.5)

MIC, Minimum Inhibitory Concentration (mg/L); S, susceptible; I, intermediate; R, resistant.

### 3.2 Antibacterial activity of dendrimers

We have tested seven dendritics compounds as antibacterials (**Figures 1**, **2**): three first-generation dendrimers with a phloroglucinol core (BDAC-001, BDLS-001 and BDEF-031), three zero-generation dendrimers with a silicon core (BDJS-049, BDJS-052 and BDSQ-017) and a second-generation dendron with a silicon core and an ibuprofen group at the focal point to combine antimicrobial and anti-inflammatory properties (BDEF-130). In addition, these compounds present different peripheral groups: ammonium (BDAC-001 and BDJS-049), guanidinium (BDLS-001 and BDJS-052) and trimethylammonium (BDEF-031, BDSQ-017 and BDEF-130).

To determine the antibacterial capabilities of the dendrimers and the dendrons against planktonic cells, MIC and MBC studies were carried out. With respect to the *S. aureus* strain ATCC 29213, the seven compounds tested showed a high *in vitro* efficacy, in a range of concentrations <1-16 mg/L, highlighting the dendrimers BDSQ-017(with trimethylammonium groups) and BDLS-001 (with guanidinium groups) and the dendron BDEF-130 (with ibuprophen at the focal point and trimethylammonium peripheral groups). A similar behaviour was observed for the clinical strains MRSA 1 and MRSA 2, which corroborates the activity of these dendrimers against resistant bacteria.

To analyse the antibiofilm activity of these compounds, MBIC and MBEC studies were performed against preformed biofilms. Since all seven compounds tested against *S. aureus* were antibacterial against planktonic cells, they were all tested on biofilms to determine their ability to inhibit and completely eradicate them. With regard to the biofilms of *S. aureus* strain ATCC 29213 (**Table 3**), it was evident that the dendrimer BDSQ-017 and the dendron BDEF-130 were able to both inhibit and eradicate them at low concentrations, in the range of 16-32 mg/L. In addition, the dendrimer BDLS-001 showed good antibiofilm activity. Again, these dendrimers showed a similar behaviour for the clinical strains MRSA 1 (**Table 4**) and MRSA 2 (**Table 5**).

**Table 3:** Antimicrobial properties of the compounds against *Staphylococcus aureus* ATCC 29213.

Strain	Compound	MIC	MBC	MBIC	MBEC
ATCC 29213	BDAC-001	16	16	128	128
	BDLS-001	<1	<1	64	64
	BDEF-031	8	8	256	256
	BDJS-049	16	16	256	256
	BDJS-052	4	4	256	256
	BDSQ-017	<1	2	32	32
	BDEF-130	<1	2	16	32

MIC, Minimum Inhibitory Concentration (mg/L); MBC, Minimum Bactericidal Concentration (mg/L); MBIC, Minimum Biofilm Inhibitory Concentration (mg/L); MBEC, Minimum Biofilm Eradication Concentration (mg/L).

**Table 4:** Antimicrobial properties of the compounds against methicillin-resistant *Staphylococcus aureus* (MRSA 1).

Strain	Compound	MIC	MBC	MBIC	MBEC
MRSA 1	BDAC-001	8	16	64	128
	BDLS-001	<1	<1	32	64
	BDEF-031	8	16	128	128
	BDJS-049	16	16	256	256
	BDJS-052	4	4	128	128
	BDSQ-017	<1	<1	16	16
	BDEF-130	<1	2	16	16

MIC, Minimum Inhibitory Concentration (mg/L); MBC, Minimum Bactericidal Concentration (mg/L); MBIC, Minimum Biofilm Inhibitory Concentration (mg/L); MBEC, Minimum Biofilm Eradication Concentration (mg/L).

**Table 5:** Antimicrobial properties of the compounds against methicillin-resistant *Staphylococcus aureus* (MRSA 2).

Strain	Compound	MIC	MBC	MBIC	MBEC
MRSA 2	BDAC-001	16	16	64	64
	BDLS-001	<1	<1	64	64
	BDEF-031	8	8	256	256
	BDJS-049	16	16	256	256
	BDJS-052	4	4	128	256
	BDSQ-017	<1	2	8	8
	BDEF-130	<1	2	16	16

MIC, Minimum Inhibitory Concentration (mg/L); MBC, Minimum Bactericidal Concentration (mg/L); MBIC, Minimum Biofilm Inhibitory Concentration (mg/L); MBEC, Minimum Biofilm Eradication Concentration (mg/L).

For the *P. aeruginosa* strain ATCC 27853 (**Table 6**), the compounds were less effective *in vitro* than against *S. aureus*. It was observed that the phloroglucinol-core dendrimers BDAC-001 (with ammonium groups) and BDLS-001 (with guanidinium groups) inhibited the bacterial growth at very low concentrations while those with silicon cores required higher concentrations. In addition, the phloroglucinol dendrimer BDEF-031 (with trymethylammonium groups), which performed well against *S. aureus*, lacked antibacterial activity against *P. aeruginosa*. On the other hand, these dendrimers performed similarly with the clinical strains PA 24 (**Table 7**) and PA 35 (**Table 8**).

**Table 6:** Antimicrobial properties of the compounds against *Pseudomonas aeruginosa* ATCC 27853.

Strain	Compound	MIC	MBC	MBIC	MBEC
ATCC 27853	BDAC-001	2	8	512	>1024
	BDLS-001	2	4	1024	>1024
	BDEF-031	1024	>1024	–	–
	BDJS-049	32	64	>1024	>1024
	BDJS-052	32	128	–	–
	BDSQ-017	64	128	–	–
	BDEF-130	64	64	>1024	>1024

MIC, Minimum Inhibitory Concentration (mg/L); MBC, Minimum Bactericidal Concentration (mg/L); MBIC, Minimum Biofilm Inhibitory Concentration (mg/L); MBEC, Minimum Biofilm Eradication Concentration (mg/L).

**Table 7:** Antimicrobial properties of the compounds against multidrug-resistant *Pseudomonas aeruginosa* (PA 24).

Strain	Compound	MIC	MBC	MBIC	MBEC
PA 24	BDAC-001	2	4	512	>1024
	BDLS-001	8	8	1024	>1024
	BDEF-031	512	1024	–	–
	BDJS-049	16	32	>1024	>1024
	BDJS-052	32	64	–	–
	BDSQ-017	32	64	–	–
	BDEF-130	16	32	>1024	>1024

MIC, Minimum Inhibitory Concentration (mg/L); MBC, Minimum Bactericidal Concentration (mg/L); MBIC, Minimum Biofilm Inhibitory Concentration (mg/L); MBEC, Minimum Biofilm Eradication Concentration (mg/L).

**Table 8:** Antimicrobial properties of the compounds against multidrug-resistant *Pseudomonas aeruginosa* (PA 35).

Strain	Compound	MIC	MBC	MBIC	MBEC
PA 35	BDAC-001	2	4	512	>1024
	BDLS-001	4	32	1024	>1024
	BDEF-031	512	>1024	–	–
	BDJS-049	16	16	>1024	>1024
	BDJS-052	32	128	–	–
	BDSQ-017	32	128	–	–
	BDEF-130	32	128	>1024	>1024

MIC, Minimum Inhibitory Concentration (mg/L); MBC, Minimum Bactericidal Concentration (mg/L); MBIC, Minimum Biofilm Inhibitory Concentration (mg/L); MBEC, Minimum Biofilm Eradication Concentration (mg/L).

Due to the lower efficacy of the compounds against the planktonic form of *P. aerugionosa*, MBIC and MBEC studies were limited to dendrimers and dendrons that exhibited superior antimicrobial activity: the dendrimers BDAC-001, BDLS-001 and BDJS-049, and the dendron BDEF-130. As can be seen in the tables, no compound was able to inhibit and eradicate the preformed biofilms of the three strains of *P. aeruginosa*.

### 3.3 Cytotoxic activity of dendrimers

To determine the toxicity and, consequently, the selectivity of the dendrimers and dendrons between bacteria and eukaryote cells, MTT viability assays were carried out on HeLa cells for those compounds with optimal antibacterial activity: the dendrimers BDAC-001, BDLS-001 and BDSQ-017, and the dendron BDEF-130. As can be seen in **Table 9**, the four compounds analized were cytotoxic at their respective IC_50_ with statistically significant differences. Based on these results, the dendrimer BDLS-001 presented a relative therapeutic margin against planktonic forms of MRSA and multidrug-resistant *P. aeruginosa*, whereas the dendrimer BDAC-001 only showed it against *P. aeruginosa*. Regarding the dendrimer BDSQ-017 and the dendron BDEF-130, they are less toxic than the phloroglucinol-core dendrimers, but showing good selectivity only against planktonic forms of MRSA.

**Table 9:** Cytotoxic activity values of the compounds on HeLa cells.

Compound	Molecular weigth (g/mol)	IC_50_ (μM)	IC_50_ (mg/L)	p-values
BDAC-001	1264.69	4.6 ± 0.4	5.9 ± 0.5	0.001
BDLS-001	1516.93	6.3 ± 0.3	9.5 ± 1.9	<0.001
BDSQ-017	1103.84	9.8 ± 0.8	10.8 ± 0.8	<0.001
BDEF-130	1571.64	6.1 ± 0.4	9.5 ± 0.7	0.001

## 4 Discussion

In this study, the antimicrobial activity of six cationic CBS dendrimers, with two different cores and three different cationic groups, and one cationic dendron (with a ibuprofen moiety at the focal point) was tested against clinical strains of multidrug-resistant bacteria and their biofilms. The results indicated that all seven compounds were effective *in vitro* against the planktonic form of the three *S. aureus* strains, but only the dendrimers BDAC-001, BDLS-001, BDSQ-017 and the dendron BDEF-130 showed antibiofilm activity *in vitro*. On the other hand, only the compounds BDAC-001, BDLS-001, BDJS-049 and BDEF-130 showed antibacterial activity against the planktonic form of the three strains of *P. aeruginosa*; although, none of them were active against the preformed biofilms.

With respect to the type of bacteria, all of them were more effective against the Gram-positive than the Gram-negative bacteria, except the dendrimer BDAC-001. This is due to the presence of an additional outer membrane in the Gram-negative bacteria, which protects them against the entry of this type of compounds. These results are in agreement with previous studies, where a slightly higher efficacy of the cationic CBS dendritic systems was observed against the planktonic forms of Gram-positive *S. aureus* than against Gram-negative *Escherichia coli* (Chen et al., 2000; Xue et al., 2013; Fuentes-Paniagua et al., 2014; Fuentes-Paniagua et al., 2016; VanKoten et al., 2016). However, other studies such as that of (Lopez et al., 2009) indicated that the PAMAM dendrimers of generations 3 and 5 were more effective *in vitro* against the Gram-negative than against the Gram-positive bacteria. It can be explained by the thick and rigid peptidoglycan layer hinders the action of these compounds because they cannot reach the bacterial plasma membrane. CBS and PAMAM dendrimers differ in hydrophobicity of the framework (much higher CBS dendrimer) and in size of dendrimers (much bigger PAMAM dendrimer). Therefore, it can be expected that modifications of the inner structure of dendrimers and of size can be useful to modulate the antibacterial action of this type of compounds.

Regarding the dendrimer core, those dendrimers with a phloroglucinol core were better as bactericides than those with a silicon atom core. Previous studies from our group such as (Fuentes-Paniagua et al., 2016) showed the same trend. This may be due to the hydrophilic-lipophilic balance of the dendrimers, represented by the peripheral groups and the CBS skeleton, respectively (Rasines et al., 2009). As the framework of CBS dendrimers is highly hydrophobic, it tends to shrink in aqueous medium to minimize its exposure to water. Nevertheless, because the phloroglucinol core is bigger and more rigid (a ring vs an atom), this type of dendrimers offers a more open structure, which could favor the interaction with bacteria membrane (Fuentes-Paniagua et al., 2016).

Regarding the cationic peripheral groups, similar activity has been observed for the three types: ammonium, guanidinium and trimethylammonium. Most studies of our group such as (Fuentes-Paniagua et al., 2016) and (Heredero-Bermejo et al., 2018) evidenced that there were no notable differences in activity of the peripheral groups against both types of bacteria, Gram-positive *S. aureus* CECT 240 and Gram-negative *E. coli* CECT 515. This may be due to the fact that the groups are of similar size and the charges are available to establish electrostatic interactions with the bacterial membranes. The study by (Fuentes-Paniagua et al., 2014) showed that dendrimers with the charge slightly hidden from the surface, -[NMe_2_(CH_2_CH_2_OH)]^+^ groups, were less active *in vitro* than those with -NH_3_^+^ and -NMe_3_^+^ groups. In addition, the study by (Heredero-Bermejo et al., 2018) described that the -NH_3_^+^ group was slightly more amoebicidal than the guanidinium group against the *Acanthamoeba polyphaga* trophozoites.

The dendrimers and the dendrons showed less activity against the biofilms than against the planktonic forms of the microorganisms. Only the dendrimers BDAC-001, BDLS-001 and BDSQ-017 and the dendron BDEF-130 inhibited and eradicated the preformed *S. aureus* biofilms, but none of them acted on those of *P. aeruginosa*. This is explained by the fact that after adhering to polysaccharides, extracellular DNA (e-DNA) and proteins in the biofilm, antimicrobials seem to be more biologically inactive or cannot reach the concentrations necessary for effective bacterial eradication (Mishra et al., 2023). In particular, the large amount of e-DNA and alginate negatively charged in biofilms of *P. aeruginosa* could neutralize these cationic compounds (Olivares et al., 2020). In addition, bacteria within biofilms exhibit altered gene expression and metabolism in response to environmental anoxia and nutrient limitation, which lead to a reduced metabolic rate and cell division. These adaptations may confer antimicrobial resistance by inactivating the antimicrobial receptor on bacteria or reducing the cellular functions that antibiotics interfere with (Abdelhamid and Yousef, 2023).

It is worth highlighting the inhibitory and eradication capacities at low concentrations of the dendrimer BDSQ-017 and the dendron BDEF-130. This could be attributed, on the one hand, to the high water-solubility of the former and on the other hand, to the permanent cationic charge of the -NMe_3_^+^ groups and the smaller size of the dendron (Sánchez-Milla et al., 2020). These factors allow these compounds to surpass better the biofilms and consequently, to have an optimal antibiofilm activity.

According to the review by (Alfei and Caviglia, 2022), there are few studies describing the potential of the dendrimers to prevent the formation of biofilms and to promote their eradication. The study by (Fuentes-Paniagua et al., 2016) established that the dendrimer BDEF-031 was able to prevent the formation of *S. aureus* CECT 240 biofilms at the concentration of 8 mg/L, but it lacked activity against preformed biofilms. Also, our results of the cationic CBS dendrimers BDAC-001, BDLS-001 and BDJS-049 and the dendron BDEF-130 on preformed *P. aeruginosa* biofilms are similar to the study by (Quintana-Sanchez et al., 2022), which showed that the dendrimer BDSQ-017 only eradicated *P. aeruginosa* CECT 108 biofilms at very high concentrations.

In order to treat bacterial infections, it is important to identify differences between antibacterial activity and toxicity in eukaryotic cells. The cytotoxicity of cationic dendrimers is due to the electrostatic interactions that they establish with the negative charges of the phosphate groups of the plasmatic membrane, which leads to the formation of nanopores and, finally, to cell death (Santos et al., 2019). This cytotoxicity depends, to a large extent, on the concentration, the generation and the number and nature of the peripheral groups, being greater for those cationic dendrimers of higher generation (Janaszewska et al., 2019; Santos et al., 2019).

In our case, the dendrimers BDAC-001, BDLS-001 and BDSQ-017 and the dendron BDEF-130 have presented *in vitro* a relative therapeutic margin, being those compounds with a silicon core more biocompatible. This may be due to the aforementioned antibacterial efficacy, i.e., having a smaller and more flexible core, these dendrimers shrink more in aqueous medium and the peripheral groups interact less well with the cell membrane, causing less cytotoxicity. The data from our study are similar to those of previous studies by our group (Perisé-Barrios et al., 2016; Heredero-Bermejo et al., 2018), Haga clic o pulse aquí para escribir texto. The study by (Heredero-Bermejo et al., 2018) demonstrated that dendrimers BDAC-001 and BDLS-001 showed optimal antibacterial and antiamebic activities *in vitro*, with a good cytotoxic profile on HeLa cells and MU-PH1 cells, with dendrimer BDLS-001 being more biocompatible because of the guanidinium peripherical groups. In addition, the study by (Perisé-Barrios et al., 2016) evidenced that compounds similar to the dendron BDEF-130 presented some cytotoxicity from 10 µM on M1 and M2 macrophages. Therefore, these compounds are possible candidates for topical antimicrobials as they exhibit an optimal antimicrobial activity and a good profile of cytotoxicity *in vitro*.

Finally, although these compounds could be applied topically, it could be important to take into account that their action can be hampered by interactions with other biomacromolecules. In this line, the dendritic systems can interact with charged protein residues by electrostatic forces. Other types of interactions have also been demonstrated, such as hydrogen bonds, van der Waals forces and hydrophobic interactions (Shcharbin et al., 2017). These bonds depend on the characteristics of both participants: the flexibility and surface charge of a dendrimer, the structural rigidity of the protein and the localization of charged amino acids on its surface. In addition, the pH and ionic strength of the solutions can significantly modulate these interactions (Shcharbin et al., 2017). This adsorption of proteins on the surface of the systems results in a “protein corona” (Shcharbin et al., 2018), which can completely changed their properties, distribution and bioavailability and is a critical challenge when attempting to use these compounds in medicine (Shcharbin et al., 2018).

Several studies have shown that cationic dendritic systems can interact with plasma proteins, such as negatively charged human serum albumin (HSA) or globulins (Shcharbin et al., 2018; Wang et al., 2018). The study by (Shcharbin et al., 2018) evidenced that gold nanoparticles modified with second and third generations dendrons, similar to the one use in this work, and their corresponding dendrons interacted significantly less with HSA than those of first generation, causing lesser effects on the structure, immunochemical properties and conformation of HSA. Moreover, the study by (Wang et al., 2018) demonstrated *in silico* that positively or negatively charged PAMAM dendrimers of generations 1-4 showed higher affinity for HSA and immunoglobulin E (IgE) than their correlated neutrally charged PAMAM dendrimers. However, also is important to note that for a preventive use as localized topical microbicides this phenomenon would be minimized.

## 5 Conclusions

In conclusion, the CBS cationic dendrimers and the dendron here analyzed were active *in vitro* against the planktonic and biofilm forms of methicillin-resistant *S. aureus*, but only against the planktonic form of multidrug-resistant *P. aeruginosa*. It is crucial to find a good hydrophilic-lipophilic balance of these compounds to optimize their antibacterial activity, since derivatives with the biggest and rigid core (the phloroglucinol core) were more active than those with the smaller core (the silicon atom core). On the other hand, apparently, the type of cationic group is not relevant for the activity of dendrimers and dendrons in these strains. Finally, further studies will be carried out in order to search for modifications in dendrimers that can be useful to surpass biofilm barrier and to reduce their cytotoxicity. These studies will allow us to characterize their biological properties and to establish their potential as possible topical antimicrobials.

## Data availability statement

The raw data supporting the conclusions of this article will be made available by the authors, without undue reservation.

## Ethics statement

The study was conducted according to the ethical requirements established by the Declaration of Helsinki. The Ethics Committee of Prıncipe de Asturias University Hospital (Alcalá de Henares, Spain) approved the study (LIB 07/2022). Due to the nature of the study, the Ethics Committee determined that no patient consent was required.

## Author contributions

All authors contributed to data analysis, drafting or revising the article, gave final approval of the version to be published, and agree to be accountable for all aspects of the work.

## References

[B1] Abdelhamid A. G. Yousef A. E. (2023). Combating bacterial biofilms: current and emerging antibiofilm strategies for treating persistent infections. Antibiotics (Basel). 12 (6), 1005. doi: 10.3390/antibiotics12061005 37370324 PMC10294981

[B2] Aguilera-Correa J. J. Fernández-López S. Cuñas-Figueroa I. D. Pérez-Rial S. Alakomi H. L. Nohynek L.. (2021). Sanguiin H-6 Fractionated from Cloudberry (Rubus chamaemorus) Seeds Can Prevent the Methicillin-Resistant Staphylococcus aureus Biofilm Development during Wound Infection. Antibiotics. 10 (12), 1481. doi: 10.3390/antibiotics10121481 34943693 PMC8698471

[B3] Alfei S. Caviglia D. (2022). Prevention and eradication of biofilm by dendrimers: A possibility still little explored. Pharmaceutics. 14 (10), 2016. doi: 10.3390/pharmaceutics14102016 36297451 PMC9610720

[B4] Barrios-Gumiel A. Sanchez-Nieves J. Pérez-Serrano J. Gómez R. de la Mata F. J. (2019). PEGylated AgNP covered with cationic carbosilane dendrons to enhance antibacterial and inhibition of biofilm properties. Int. J. Pharm. 569, 118591. doi: 10.1016/j.ijpharm.2019.118591 31394187

[B5] Ceri H. Olson M. E. Stremick C. Read R. R. Morck D. Buret A. (1999). The calgary biofilm device: new technology for rapid determination of antibiotic susceptibilities of bacterial biofilms. J. Clin. Microbiol. 37 (6), 1771–1776. doi: 10.1128/JCM.37.6.1771-1776.1999 10325322 PMC84946

[B6] Chen C. Z. Beck-Tan N. C. Dhurjati P. van Dyk T. K. LaRossa R. A. Cooper S. L. (2000). Quaternary ammonium functionalized poly(propylene imine) dendrimers as effective antimicrobials: structure–activity studies. Biomacromolecules. 1 (3), 473–480. doi: 10.1021/bm0055495 11710139

[B7] CLSI (2018). “Methods for dilution antimicrobial susceptibility tests for bacteria that grow aerobically,” in CLSI standard M07, 11th ed (Wayne, PA: Clinical and Laboratory Standards Institute).

[B8] Costerton J. W. Stewart P. S. Greenberg E. P. (1999). Bacterial biofilms: A common cause of persistent infections. Science. 284 (5418), 1318–1322. doi: 10.1126/science.284.5418.1318 10334980

[B9] Dadgostar P. (2019). Antimicrobial resistance: implications and costs. Infect. Drug Resist. 12, 3903–3910. doi: 10.2147/IDR.S234610 31908502 PMC6929930

[B10] de la Mata F. J. Gómez R. Cano J. Sánchez-Nieves J. Ortega P. Gallego S. G. (2023). Carbosilane dendritic nanostructures, highly versatile platforms for pharmaceutical applications. Wiley Interdiscip. Rev. Nanomed. Nanobiotechnol. 15 (3), e1871. doi: 10.1002/wnan.1871 36417901

[B11] Fuentes-Paniagua E. Hernández-Ros J. M. Sánchez-Milla M. Camero M. A. Maly M. Pérez-Serrano J.. (2014). Carbosilane cationic dendrimers synthesized by thiol–ene click chemistry and their use as antibacterial agents. RSC Adv. 4 (3), 1256–1265. doi: 10.1039/C3RA45408H

[B12] Fuentes-Paniagua E. Sánchez-Nieves J. Hernández-Ros J. M. Fernández-Ezequiel A. Soliveri J. Copa-Patiño J. L.. (2016). Structure–activity relationship study of cationic carbosilane dendritic systems as antibacterial agents. RSC Adv. 6 (9), 7022–7033. doi: 10.1039/C5RA25901K

[B13] Gholami M. Mohammadi R. Arzanlou M. Akbari Dourbash F. Kouhsari E. Majidi G.. (2017). *In vitro* antibacterial activity of poly (amidoamine)-G7 dendrimer. BMC Infect. Dis. 17 (1), 395. doi: 10.1186/s12879-017-2513-7 28583153 PMC5460590

[B14] Gil-Gil T. Laborda P. Sanz-García F. Hernando-Amado S. Blanco P. Martínez J. L. (2019). Antimicrobial resistance: A multifaceted problem with multipronged solutions. Microbiologyopen. 8 (11), e945. doi: 10.1002/mbo3.945 31724836 PMC6855134

[B15] Heredero-Bermejo I. Hernández-Ros J. M. Sánchez-García L. Maly M. Verdú-Expósito C. Soliveri J.. (2018). Ammonium and guanidine carbosilane dendrimers and dendrons as microbicides. Eur. Polym J. 101, 159–168. doi: 10.1016/j.eurpolymj.2018.02.025

[B16] Heredero-Bermejo I. Sánchez-Nieves J. Soliveri J. Gómez R. de la Mata F. J. Copa-Patiño J. L.. (2016). *In vitro* anti- Acanthamoeba synergistic effect of chlorhexidine and cationic carbosilane dendrimers against both trophozoite and cyst forms. Int. J. Pharm. 509 (1–2), 1–7. doi: 10.1016/j.ijpharm.2016.04.075 27173821

[B17] Hernandes C. Ceppede J. Bertoni B. França S. Pereira A. (2013). Flash microbiocide: A rapid and economic method for determination of MBC and MFC. Am. J. Plant Sci. 4 (4), 850–852. doi: 10.4236/ajps.2013.44104

[B18] Janaszewska A. Lazniewska J. Trzepiński P. Marcinkowska M. Klajnert-Maculewicz B. (2019). Cytotoxicity of dendrimers. Biomolecules. 9 (8), 330. doi: 10.3390/biom9080330 31374911 PMC6723213

[B19] Kumar P. Nagarajan A. Uchil P. D. (2018). Analysis of cell viability by the MTT assay. Cold Spring Harb. Protoc. 2018 (6). doi: 10.1101/pdb.prot095505 29858338

[B20] Lopez A. I. Reins R. Y. McDermott A. M. Trautner B. W. Cai C. (2009). Antibacterial activity and cytotoxicity of PEGylated poly(amidoamine) dendrimers. Mol. Biosyst. 5 (10), 1148. doi: 10.1039/b904746h 19756304 PMC2965593

[B21] Mishra S. Gupta A. Upadhye V. Singh S. C. Sinha R. P. Häder D. P. (2023). Therapeutic strategies against biofilm infections. Life (Basel) 13 (1), 172. doi: 10.3390/life13010172 36676121 PMC9866932

[B22] Nam-Cha S. H. Domínguez-Jurado E. Tinoco-Valencia S. L. Pérez-Tanoira R. Morata-Moreno N. Alfaro-Ruiza R.. (2023). Synthesis, characterization, and antibacterial activities of a heteroscorpionate derivative platinum complex against methicillin-resistant Staphylococcus aureus. Front. Cell Infect. Microbiol. 13. doi: 10.3389/fcimb.2023.1100947 PMC10083354 37051297

[B23] Olivares E. Badel-Berchoux S. Provot C. Prévost G. Bernardi T. Jehl F. (2020). Clinical impact of antibiotics for the treatment of pseudomonas aeruginosa biofilm infections. Front. Microbiol. 10. doi: 10.3389/fmicb.2019.02894 PMC6962142 31998248

[B24] Ortega P. Sánchez-Nieves J. Cano J. Gómez R. de la Mata F. J. (2020) p, 114–145.

[B25] Parsek M. R. Greenberg E. P. (2005). Sociomicrobiology: the connections between quorum sensing and biofilms. Trends Microbiol. 13 (1), 27–33. doi: 10.1016/j.tim.2004.11.007 15639629

[B26] Perisé-Barrios A. J. Fuentes-Paniagua E. Sánchez-Nieves J. Serramía M. J. Alonso E. Reguera R. M.. (2016). Improved efficiency of ibuprofen by cationic carbosilane dendritic conjugates. Mol. Pharm. 13 (10), 3427–3438. doi: 10.1021/acs.molpharmaceut.6b00420 27533491

[B27] Pettit R. K. Weber C. A. Kean M. J. Hoffmann H. Pettit G. R. Tan R.. (2005). Microplate Alamar blue assay for Staphylococcus epidermidis biofilm susceptibility testing. Antimicrob. Agents Chemother. 49 (7), 2612–2617. doi: 10.1128/AAC.49.7.2612-2617.2005 15980327 PMC1168683

[B28] Quintana-Sanchez S. Gómez-Casanova N. Sánchez-Nieves J. Gómez R. Rachuna J. Wąsik S.. (2022). The antibacterial effect of PEGylated carbosilane dendrimers on P. aeruginosa alone and in combination with phage-derived endolysin. Int. J. Mol. Sci. 23 (3), 1873. doi: 10.3390/ijms23031873 35163794 PMC8836974

[B29] Rasines B. Hernández-Ros J. M. de las Cuevas N. Copa-Patiño J. L. Soliveri J. Muñoz-Fernández M. A.. (2009). Water-stable ammonium-terminated carbosilane dendrimers as efficient antibacterial agents. Dalton Trans. 40), 8704–8713. doi: 10.1039/b909955g 19809746

[B30] Sánchez-Milla M. Gómez R. Pérez-Serrano J. Sánchez-Nieves J. de la Mata F. J. (2020). Functionalization of silica with amine and ammonium alkyl chains, dendrons and dendrimers: Synthesis and antibacterial properties. Materials Sci. Engineering: C. 109, 110526. doi: 10.1016/j.msec.2019.110526 32228896

[B31] Santos A. Veiga F. Figueiras A. (2019). Dendrimers as pharmaceutical excipients: synthesis, properties, toxicity and biomedical applications. Materials (Basel). 13 (1), 65. doi: 10.3390/ma13010065 31877717 PMC6981751

[B32] Shcharbin D. Pedziwiatr-Werbicka E. Serchenya T. Cyboran-Mikolajczyk S. Prakhira L. Abashkin V.. (2018). Role of cationic carbosilane dendrons and metallic core of functionalized gold nanoparticles in their interaction with human serum albumin. Int. J. Biol. Macromol. 118 (Pt B), 1773–1780. doi: 10.1016/j.ijbiomac.2018.07.023.37 29997045

[B33] Shcharbin D. Shcharbina N. Dzmitruk V. Pedziwiatr-Werbicka E. Ionov M. Mignani S.. (2017). Dendrimer-protein interactions versus dendrimer-based nanomedicine. Colloids Surf B Biointerfaces. 152, 414–422. doi: 10.1016/j.colsurfb.2017.01.041 28167455

[B34] VanKoten H. W. Dlakic W. M. Engel R. Cloninger M. J. (2016). Synthesis and biological activity of highly cationic dendrimer antibiotics. Mol. Pharm. 13 (11), 3827–3834. doi: 10.1021/acs.molpharmaceut.6b00628 27661609 PMC9389851

[B35] Verderosa A. D. Totsika M. Fairfull-Smith K. E. (2019). Bacterial biofilm eradication agents: A current review. Front. Chem. 28. doi: 10.3389/fchem.2019.00824 PMC6893625 31850313

[B36] Wang B. Sun Y. Davis T. P. Ke P. C. Wu Y. Ding F. (2018). Understanding effects of PAMAM dendrimer size and surface chemistry on serum protein binding with discrete molecular dynamics simulations. ACS Sustain Chem. Eng. 6 (9), 11704–11715. doi: 10.1021/acssuschemeng.8b01959 30881771 PMC6413314

[B37] Xue X. Chen X. Mao X. Hou Z. Zhou Y. Bai H.. (2013). Amino-terminated generation 2 poly(amidoamine) dendrimer as a potential broad-spectrum, nonresistance-inducing antibacterial agent. AAPS J. 15 (1), 132–142. doi: 10.1208/s12248-012-9416-8 23135925 PMC3535096

